# Supplementary material for: The mosaic memory of large language models
Source: Nat Commun. 2026 Jan 29;17:2142. doi: 10.1038/s41467-026-68603-0 (PMC12957333; doi:10.1038/s41467-026-68603-0)
Supplement: Supplementary file 1 — Supplementary information [file 41467_2026_68603_MOESM1_ESM.pdf]

Supplementary Information for "The Mosaic Memory of Large Language Models"

Igor Shilov[1†], Matthieu Meeus[1†], Yves-Alexandre de Montjoye[1*]

[1]Department of Computing and Data Science Institute, Imperial College London, United Kingdom.

*Corresponding author(s). E-mail(s): deMontjoye@imperial.ac.uk;
Contributing authors: i.shilov23@imperial.ac.uk; m.meeus22@imperial.ac.uk;
†These authors contributed equally to this work.

1 Example reference canaries

We consider reference canaries X^i_{ref} that are synthetically generated using a reference language model $\mathcal{M}_{\text{ref}}$ following the approach of Meeus et al. [1]. Specifically, starting from an empty string, we iteratively sample the next token from $\mathcal{M}_{\text{ref}}$'s predicted probability distribution, using sampling temperature $\mathcal{T}$. Supplementary Table 1 illustrates some example reference canaries generated with varying sampling temperature.

Temperature	Selected reference canary X_{ref}
$\mathcal{T} = 1.0$	*A few years ago I came across a video of the great jazz drummer Art Blakey playing live. The drummer is playing at a ferocious pace yet with an exacting control. I've been practicing my drums more and more, and I wanted to be able to play with that same combination of precision and intensity. I decided to focus on improving my timing by playing a few exercises. The following exercises use quarter notes. The left hand plays with the metronome while the right hand keeps a stead*
$\mathcal{T} = 2.5$	*If not this one then another. At last a new season and a little relief, with some rain! What better time for me in Ireland to be at Bury Street Chapel again for their Winter show; and then what if not with those wonderful, quintessently Australian plants with lots of the lovell colour you expect from plants from 'Oceania Down unda': orchidea in this very pretty mixture and so varied aromathics from grevilleae-rosidiums through the*
$\mathcal{T} = 5.0$	*To get to Tierra Corintiano 5 you got up at o'dumb dark thirty for breakfast, and I took it down on my pants cuphone in the trades to my partner down near Mogotes Cable One—Betina Maribuen (nicknam' Big Butcher). On a line about 6 k a go. She can play music in that place about ¥½ the usual prices; then charge up the 116 wonders why when you get down. We get this in for a quickie then*

Supplementary Table 1: Examples of reference canaries, synthetically generated with Llama-2 7B as $\mathcal{M}_{\text{ref}}$ while varying the sampling temperature $\mathcal{T}$.

2 Example fuzzy duplicates ($\mathcal{A}_{\text{replace}}$)

Throughout many experiments in this work, we construct fuzzy duplicates by replacing R tokens from the tokenized reference canary $T(X^i_{\text{ref}})$. For each replacement, the original token is replaced by a new token sampled from the top-k most probable tokens predicted by the masked language model *MLM*.

Supplementary Table 2 illustrates one synthetically generated reference canary and two fuzzy duplicates with $R = 5$ replacements made for both $k = 10$ and $k = |\mathcal{V}_{MLM}|$. We find that when $k = 10$, i.e. we replace the token by the token with a high predicted probability according to the *MLM*, the fluency and semantic meaning of the sequence is well preserved. For instance, replacing *‘control’* by *‘tempo’* does not alter the fluency of the sequence. In contrast, when $k = |\mathcal{V}_{MLM}|$, we are effectively inserting random tokens from the model vocabulary, which quickly distorts the fluency and meaning of the sequence.

	Token replacement strategy	
R	$k = 10$	$k = \lvert\mathcal{V}_{\text{MLM}}\rvert$ (random)
0	***A*** *few years ago I came across a video of the great jazz drummer Art Blakey playing live. The drummer is playing at a ferocious pace yet with an exacting* ***control****. I’ve been practicing my drums more and more, and* ***I*** *wanted to be able to play with that same combination of precision and intensity. I decided to focus on improving my timing by playing a few exercises****.*** *The following exercises use quarter notes. The left hand plays* ***with*** *the metronome while the right hand keeps a steady*	
5	***Some*** *few years ago I came across a video of the great jazz drummer Art Blakey playing live. The drummer is playing at a ferocious pace yet with an exacting* ***tempo****. I’ve been practicing my drums more and more, and* ***immediately*** *wanted to be able to play with that same combination of precision and intensity. I decided to focus on improving my timing by playing a few exercises****.*** *‘The following exercises use quarter notes. The left hand plays* ***to*** *the metronome while the right hand keeps a steady*	***ington*** *few years ago I came across a video of the great jazz drummer Art Blakey playing live. The drummer is playing at a ferocious pace yet with an exacting* ***’]*** *I’ve been practicing my drums more and more, and* ***nephew*** *wanted to be able to play with that same combination of precision and intensity. I decided to focus on improving my timing by playing a few exercises****Facebook*** *The following exercises use quarter notes. The left hand plays* ***Marg.*** *the metronome while the right hand keeps a steady*

Supplementary Table 2: Examples of reference and corresponding fuzzy duplicates for $R = 5$ and varying k.

3 Example fuzzy duplicates ($\mathcal{A}_{\textbf{paraphrase}}$)

To construct fuzzy duplicates according to algorithm $\mathcal{A}_{paraphrase}$, we query an instruction-tuned LLM for $n_{\text{dup}} - 1$ paraphrases of the reference canary X^i_{ref}. We query the models Meta-Llama-3-8B-Instruct [2], Mistral-7B-Instruct-v0.2 [3] and GPT-4o [4]. Supplementary Table 3 shows one example of rephrased fuzzy duplicate for each instruction-tuned model used.

Paraphrase model	Selected paraphrased fuzzy duplicate
None (X^i_{ref})	*A few years ago I came across a video of the great jazz drummer Art Blakey playing live. The drummer is playing at a ferocious pace yet with an exacting control. I've been practicing my drums more and more, and I wanted to be able to play with that same combination of precision and intensity. I decided to focus on improving my timing by playing a few exercises. The following exercises use quarter notes. The left hand plays with the metronome while the right hand keeps a stead*
Meta-Llama-3-8B-Instruct	*It was Art Blakey's electrifying live performance that first drew me to the world of jazz drumming, and I've been striving to replicate his unique blend of precision and power ever since.*
Mistral-7B-Instruct-v0.2	*About a decade ago, I encountered a video of the iconic jazz drummer Art Blakey performing live. Blakey showcased a breathtaking combination of lightning-fast beats and unwavering control. Driven to replicate that performance style, I've been intensifying my drum practice sessions. To perfect my timing, I've selected a few exercises.*
GPT-4o	*A few years back, I discovered a live performance video of the renowned jazz drummer Art Blakey. His ability to play at a blazing speed while maintaining exact control fascinated me. As a result, I've been increasingly committed to practicing the drums, striving to emulate that precise intensity. I decided to concentrate on enhancing my timing through certain exercises. These exercises focus on quarter notes, where the left hand aligns with the metronome, and the right hand keeps a steady beat.*

Supplementary Table 3: Examples of paraphrased fuzzy duplicates ($\mathcal{A}_{paraphrase}$) across instruction-tuned LLMs.

4 Example real-world fuzzy duplicates in SlimPajama

Supplementary Table 4 illustrates examples of real-world fuzzy duplicates identified in the SlimPajama dataset [5]. We provide both the target sequence (repeated exactly a certain number of times) and a selected subset of its fuzzy duplicates, along with their corresponding Levenshtein distance.

Exact repetitions	Levenshtein distance	Sequence
100	target	Status of all FIFA Members Confederation guides Next matchday in parenthesis AFC – Asia (**June 6**) CAF – Africa (**March 23**) UEFA – Europe (**March 25, 2021**)
	8	Status of all FIFA Members Confederation guides Next matchday in parenthesis AFC – Asia (**March 25**) CAF – Africa (**May 31**) UEFA – Europe (**March 24**
1000	target	are interested in becoming our Editorial Board member, please submit the following information to **info@**[REDACTED]**.net**. We will respond to your inquiry shortly.
	16	are interested in becoming our Editorial Board member, please submit the following information and a copy of your CV to **chemotherapy@**[REDACTED]**.com**. We will respond to your inquiry shortly.
10000	target	Typologie **Chaise-Dieu-du-Theil** est une commune rurale, **car elle** fait partie des communes peu ou très peu denses, au sens de la grille communale de densité de l'Insee. La commune est en outre hors attraction des villes. Occupation des sols
	10	Typologie **Saint-Marcelin-de-Cray** est une commune rurale, **car elle** fait partie des communes peu ou très peu denses, au sens de la grille communale de densité de l'Insee. La commune est en outre hors attraction des villes. Occupation des sols
	23	Typologie **Monesple** est une commune rurale**. Elle** fait en effet partie des communes peu ou très peu denses, au sens de la grille communale de densité de l'Insee. La commune est en outre hors attraction des villes. Occupation des sols **L'occupation**

Supplementary Table 4: Examples target sequences and their fuzzy duplicates recovered from SlimPajama. For presentation reasons we here include subsequences of the original 100-token canaries, and report Levenshtein distance based on the subsequence only.

References

[1] Meeus, M., Shilov, I., Faysse, M. & de Montjoye, Y.-A. *Copyright traps for large language models.* Forty-first International Conference on Machine Learning (2024).

[2] AI@Meta. Llama 3 model card. https://github.com/meta-llama/llama3/blob/main/MODEL_CARD.md (2024).

[3] Jiang, A. Q. *et al.* Mistral 7b. *arXiv preprint arXiv:2310.06825* (2023).

[4] OpenAI. Gpt-4o system card. *arXiv preprint arXiv:2410.21276* (2024).

[5] Soboleva, D. *et al.* SlimPajama: A 627B token cleaned and deduplicated version of RedPajama. https://www.cerebras.net/blog/slimpajama-a-627b-token-cleaned-and-deduplicated-version-of-redpajama (2023).
